# Future of anti-VEGF: biosimilars and biobetters

**DOI:** 10.1186/s40942-021-00343-3

**Published:** 2022-01-04

**Authors:** Monika Kapur, Suvansh Nirula, Mayuresh P. Naik

**Affiliations:** 1 Department of Ophthalmology, H.I.M.S.R. & H.A.H.C. Hospital, Hamdard University, Room No.3 of Eye OPD, First Floor of New OPD Building, H.A.H.C. Hospital, near GK-2, Alaknanda, Delhi 110062 New Delhi, India; 2 Department of Ophthalmology, H.I.M.S.R & H.A.H.C Hospital, near GK-2, Alaknanda, New Delhi 110062 India; 3 Oxford Eye Hospital, John Radcliffe Hospital, Oxford University Hospitals NHS Trust, Headley way, Headington, OX3 9DU Oxford UK

**Keywords:** Biosimilars, Biobetters, Anti-VEFG

## Abstract

The advent of Anti- VEGFs like Lucentis (Ranibizumab), Eylea (Aflibercept) and off-label Avastin (Bevacizumab) have radically improved visual outcomes in patients of neovascular Age Related Macular Degeneration (nARMD), Diabetic Macular Edema (DME) and Retinal Vein Occlusion (RVO). It is a matter of great concern that the US patents for Ranibizumab and Aflibercept expired in 2020 with European patents to expire in 2022 and 2025, respectively. With the expiry of these biologics, Biosimilars can prove to be saviours in the posterior segment pharmacotherapy owing to their cost effectiveness and availability of various options. Numerous biosimilars are expected to gain approval for clinical use from the US-FDA and EMA soon. Biobetters are better than the original biologic in one or more parameters but require more research and development resources. With the emergence of better manufacturing and purification processes it is imperative that the biologics and biosimilars become better. The Ophthalmologists need to have in depth knowledge about these Biosimilars and Biobetters before these molecules take over the mainstream market.

## Introduction

There have been considerable advances in the field of posterior segment pharmacotherapy since researchers developed anti-VEGF molecules like Lucentis® (Ranibizumab), Eylea® (Aflibercept), and off-label Bevacizumab (Avastin). These biologics have proved to be the cause for immense financial burden in the eyecare sector. The exorbitant cost and unfulfilled expectations of visual recovery have contributed to patients' early discontinuation of treatment. A concerning fact is that the U.S. patents for Bevacizumab and Ranibizumab expired in 2019 and 2020, respectively, while that for Aflibercept expires in 2023. The European patents of these drugs are to expire in 2022 for Bevacizumab and Ranibizumab and 2025 for Aflibercept [1]. With the expiry of these patents, biosimilars can prove to be a suitable and cheaper alternative. This transition to biosimilars can make a substantial impact all over the world. The overall understanding of biosimilars for ophthalmological purposes is essential before they are available for clinical use worldwide.

The anti- VEGFs are given intravitreally to treat numerous retinal disorders such as macular edema due to wet Age-related Macular Degeneration (AMD), Diabetic Macular Edema, and Retinal Vein Occlusion (RVO). The purpose of this review would be to provide the science behind biosimilars, the ones that are showing positive results, and how with developing technology, they can impact the world of ophthalmology.

According to the World Health Organization (WHO), Biosimilars are defined as biotechnological products that are comparable with an already approved reference product in quality, nonclinical, and clinical evaluation [2]. A biosimilar molecule should demonstrate similarities in pharmacokinetics, pharmacodynamics, safety, and efficacy to the innovator biologic.

It is important to note that there is a considerable difference between generics and biosimilars. It is easy to synthesize generic drugs by matching the formula, whereas biosimilars require living cells during manufacturing. Generic drugs are stable as their composition and synthesis process is predefined but, biosimilars need to be given much attention in terms of their stability. Immunogenicity is a significant issue concerning biosimilars as living cells are used, and the manufacturing process is different for different developers [3]. It is not simple to manufacture biosimilars as they are not produced based on a predefined formulation because the original biological producing firms cannot provide the complete set of information regarding the process followed while synthesizing the drugs. Manufacturers of biosimilars must proceed with partial, incomplete information and reverse engineer the original biologic. The investment and the research required to manufacture biosimilars are considerably higher than the development of generic drugs [3]. Even though making biosimilar is exceptionally challenging, there is a race going on to develop them. The main reason behind this is the estimated profit. The time and money required to manufacture a biosimilar are much less as compared to original biologics. It is estimated that for typical biologics, 10–15 years are required to develop with an investment of around 1200–1500 million USD, whereas biosimilars can be produced in 8–10 years at a fraction of the cost mentioned above [4, 5]. This situation can easily be explained because biosimilars do not require heavy investments in clinical trials and instead use more straightforward and robust analytical techniques.

No fixed guidelines have yet been established to approve a Biosimilar, and these are different in different countries. They are constantly changing with time and technology. The basic concepts remain analysis to establish bio similarity, toxicity assessment through animal studies, and a clinical study to understand the biosimilar's safety, efficacy, and Immunogenicity.

## Biosimilars with favourable results

### Biosimilar to Ranibizumab

A total of ten manufacturers are working on Ranibizumab biosimilar, some of these are approved, and others are still in the development stage [6]. Apart from Razumab, a product of Intas Pharmaceuticals Ltd., which is approved in India, Byooviz (SB11), from Samsung Bioepis, South Korea, has recently been approved by US-FDA and EMA [7]. six other biosimilars, namely FYB 201(Germany), Xlucane (Sweden), R-TPR-024(India), SJP-0133(Japan), LUBT010(India), and CKD-701 (South Korea) are in advanced stages of clinical trials (Table 1).

**Table 1:** Biosimilars of ranibizumab

Name of Biosimilar	Manufacturer	Status
Razumab	Intas Pharmaceuticals Ltd., India	Approved by DGCI in 2015
Ranizurel/ R-TPR-024	Reliance Life Sciences, India	Approved by DGCI in 2020
SB11 Byooviz	Samsung Bioepis, South Korea	US FDA (2021), EMA (2021)
FYB201	Formycon AG/ Bioeq	BLA review accepted by FDA
Xlucane	Xbrane Biopharma, Sweden	Phase 3 trial active
SJP-0133/GBS-007	Senju Pharmaceutical, Japan	Phase 3 trial completed; results awaited
LUBT010	Lupin Ltd., India	Phase 3 trial active; recruitment completed
CKD-701	Chong Kun Dang, South Korea	Phase 3 trial completed
PF582	Pfenex, USA	Phase1/2 trial completed; on hold
BCD100	BIOCND, South Korea; Qilu Pharmaceuticals, China	Phase 3 clinical trial active; recruiting

Razumab® (Intas Pharmaceuticals Ltd., Ahmedabad, India) is the first biosimilar to Ranibizumab. It was approved in India in 2015 and has been in clinical use since then. This approval was granted based on the outcomes of phase 3 clinical trials. A total of 104 Indian patients with wet AMD participated in this trial. Along with this, Intas Pharmaceuticals also conducted a RE-ENACT study, a retrospective pooled study to assess and evaluate the experience of Razumab in 561 Indian patients with diseases like wet AMD, Retinal vein occlusion, and Diabetic Macular Edema [8, 9]. Another study, i.e., the Clinical Efficacy and Safety of Razumab® (CESAR) study, which was a retrospective analysis with an independent framework as opposed to the ones conducted by Intas pharmaceuticals, was done to evaluate the utilization of Razumab® in Indians with chorioretinal vascular diseases such as DME, choroidal neovascular membrane (CNVM), and macular edema secondary to retinal vein occlusion. The CESAR study suggested an improvement in visual acuity after Razumab® injection detected as early as one month. In 121 treatment naïve eyes, there was a significant improvement in mean CDVA from baseline to month 3 in each subgroup (DME, CNVM, and RVO). The mean % improvement in CFT was 37% in naïve DME eyes, 26.7% in naïve CNVM eyes, and 39.6% in naïve RVO eyes at the end of 3 months. There was no evidence of toxicity or Immunogenicity after the intravitreal administration of biosimilar Ranibizumab. No patient had an intraocular pressure of more than 20 mmHg on day 1. Along with this, no systemic adverse events were reported up to 3 months follow-up [10].

The findings of the CESAR study were consistent with the data reported by the RE-ENACT study and Sameera et al. [11–16]. The RE-ENACT study showed a significant improvement in the visual acuity and central macular thickness and intra- and subretinal fluid in patients (n = 561) with wet AMD, DME, and RVO over 12 weeks [11–13]. The RE-ENACT 2 (n = 341) study evaluated the same numbers for a longer-term (48 weeks) in patients with wet AMD, DME, RVO, along with patients with myopic CNVM [13–16]. In the RE-ENACT study, three Razumab® injections were given whereas, the RE-ENACT 2 (n = 341) study evaluated patients who received one to five biosimilar Ranibizumab injections. Both studies resulted in no serious adverse events. Sameera et al. conducted a prospective study in a total of 95 Indian patients with wet AMD, DME, and RVO for the evaluation of the safety and efficacy of Razumab® over one month. The study results suggested that the biosimilar improved the visual acuity and central macular thickness. The study also did not result in any signs of ocular or systemic toxicity [11].

The biosimilar has been an enormous success, with the number of units sold in 2018 matching the sale of the original biologic (unpublished data from the manufacturer). Initially, some adverse drug reactions such as sterile endophthalmitis raised concerns about safety after the biosimilar launch. The cause was identified to be higher endotoxin levels in the buffer used for manufacturing. However, it was reported only for specific batches. The fact that these occurrences took place in clusters puts a big question mark on quality control. Consequently, manufacturing was stopped and refined, after which batches were released slowly along with careful monitoring. The drug has proven to be safe, efficacious, and approved for all indications in which Ranibizumab is used [17, 18].

SB11 Byooviz (Samsung Bioepis, South Korea) is the first ophthalmology biosimilar approved by US-FDA in September 2021 to treat wet AMD, Macular Edema following RVO, and myopic CNVM, and it attained marketing authorization by EMA in August 2021. 7 A randomized phase 3 multicentre, parallel-group double-masked study compared efficacy, safety, pharmacokinetics & Immunogenicity of SB11 with the reference Ranibizumab in patients of nAMD. 705 patients were enrolled and randomized (1:1) to receive SB11 or reference Ranibizumab every 4 weeks through week 48. The Least Squares (L.S.) mean change in best-corrected visual acuity (BCVA) from baseline at week 52 was 9.79 letters for SB11 vs. 10.41 letters for Lucentis [difference: − 0.62, (90% CI: − 2.092, 0.857)]. The LS mean change in central subfield thickness (CST) was − 139.55 μm for SB11, compared with − 124.46 μm for reference ranibizumab [difference: − 15.09, (95% CI, − 25.617, − 4.563)]. The safety and immunogenicity profile of SB11 and reference ranibizumab were comparable at all points up to week 52 [19].

FYB201 (Formycon AG and bioeq GmbH, Germany) Currently, the results of the clinical phase 3 trial (COLUMBUS-AMD) that was initiated in 2016 have been satisfactory, with BCVA and side effects comparable to Ranibizumab. The study aimed to prove the comparability of FYB201 with Lucentis® in patients with neovascular age-related macular degeneration considering the safety, efficacy, and Immunogenicity. The study achieved its primary endpoint in May 2018 and was conducted successfully, with the results suggesting that the efficacy of FYB201 is comparable to that of Lucentis® in neovascular age-related macular degeneration. Along with biosimilar, the pharmaceutical company is also developing an application system for its administration [20]. The US-FDA has accepted for reviewing the BLA for FYB201 and assigned a target action date of August 2022 [21].

Xlucane (Xbrane Biopharma, Sweden) The manufacturers have a patented technology that may yield better results than the original technology used. In vitro comparative studies where the proteins along five main dimensions were compared: amino acid sequence, folding of the protein, binding with the growth factor VEGF, biological activity, and purity. Similarity with original biologic was demonstrated on most testing aspects except for purity, which was lower than the reference drug. This is being addressed through a few modifications in the purification process during the scale-up of the production process. Currently, the XPLORE trial is taking place, a phase III multicenter, double-masked, randomized, parallel-group study in subjects with nAMD. A total of 580 patients are listed as participants and randomized to receive either Lucentis® or Xlucane in a 1:1 ratio. Xlucane is being administered in the study eye (that fits the enrollment criteria) once every four weeks for 52 weeks. The study is estimated to be completed by December 2021 [22]. According to the interim six months results, Xlucane met the primary endpoint and demonstrated equivalent efficacy in BCVA as compared to the original biologic at 8 weeks. Xbrane plans to submit BLA to Us-FDA and Marketing Authorization Application to EMA in Q4 and Q3 of 2021, respectively, based on interim results [23].

R-TPR-024/Ranizurel (Reliance Life Sciences Pvt Ltd, India) completed the phase 3 trial in Nov 2019. A total of 159 patients of nAMD were recruited and continued to receive either R-TPR-024 or innovator ranibizumab (randomised1:1) every four weeks for 24 weeks with a follow-up period of 6 months. The evaluation of efficacy, safety, and Immunogenicity showed no clinically significant difference for biosimilar ranibizumab with the reference product [24]. DGCI approved it in 2020 [25].

SJP-0133/GBS-007 (Senju Pharmaceutical, Japan) is under phase 3 trial and is expected to complete the study by 2022. 338 patients are recruited in the study, and after 1:1 randomization, three intravitreal injections of SJP-0133 or original biologic will be given once every four weeks till eight weeks and after that pro-re-nata (PRN) injections from week 12 to week 48. SJP-0133 is the only biosimilar that has included PRN regimen in the trial [26].

LUBT010 (LUPIN Ltd., India) entered a phase 3 trial in March 2019, where 200 patients were given an injection every month for three months, and results were analysed at the end of 3 months. The manufacturers are aiming for a global trial where 656 patients are to be recruited. In the global trial, patients will receive an injection every four weeks for 48 weeks. The primary outcome, i.e., BCVA, will be measured at eight weeks. The study is scheduled to be completed in October 2022 [27].

CKD-701 (Chong Kun Dang, South Korea) has completed phase 3 clinical trial in March 2021. 312 subjects were recruited and randomized 1:1 to receive either CKD-701 or Lucentis once a month during the loading phase of 3 months, and for the next 9 months, subjects received an injection on interval of a month based on the criteria in the PRN phase [28]. The company plans to submit results for domestic approval in the latter half of the year [29].

### Biosimilar to Aflibercept

MYL-1701P (Momenta Pharmaceuticals and Mylan NV, USA) A randomized, double-blinded, active control, multicenter study with 324 suitable patients with diabetes mellitus and central DME completed in September 2021. The study involved a 1:1 randomization for intravitreal treatment with MYL-1701P or Eylea®. Participants received the treatment until Week 48. All participants were evaluated for safety, Efficacy, Pharmacokinetics, and Immunogenicity of the drug [30]. The manufacturer plans to apply for US-FDA in 2022 and seeks marketing approval in the USA by 2023 [31] (Table 2).

**Table 2:** Biosimilars of aflibercept

Name of Biosimilar	Manufacturer	Status
MYL-1701P	Momenta Pharmaceuticals and Mylan NV, USA	Phase 3 trial completed
ABP-938	Amgen, USA	Phase 3 trial active; recruitment stage
FYB203	Formycon AG/Bioeq, Germany	Phase 3 trial active; recruitment stage
SB-15	Samsung Bioepis Co. Ltd, South Korea	Phase 3 trial active; recruitment stage
SOK583A19	Sandoz, Switzerland	Phase 3 trial active; recruitment stage
CT-P42	Celltrion, South Korea	Phase 3 trial active; recruitment stage
ALT-L9	Alteogen, South Korea	Phase 1 trial begun; not yet recruiting
OT-702	Ocumension Therapeutics/Shandong Boan Biological Technology, China	Phase 3 trial active

ABP-938 (Amgen, USA) is under the phase 3 trial. A randomized multicentric trial with 566 patients of nAMD was included. These patients will receive either injection ABP-938 or innovator aflibercept (randomized1:1) every 8 weeks. The subjects receiving Aflibercept will again be randomized 1:1 at 16 weeks, with 50% of patients being switched to ABP-938 injection. The patients will receive injections every 8 weeks for 48 weeks and follow up till 52 weeks. The study is scheduled to be completed by July 2023 [32].

FYB203 (Formycon AG/Bioeq, Germany) has entered phase 3 clinical trial (MAGELLAN-AMD), which started in March 2020 and is expected to be completed by August 2022. It is a randomized, double-masked, multicenter study to compare the efficacy and safety of the FYB203 compared to innovator aflibercept in terms of safety, efficacy, and Immunogenicity in patients with nAMD. The company has started recruitment of 400 patients. Patients will receive one intravitreal injection of FYB203 every 4 weeks for the first 3 doses, followed by one intravitreal injection every 8 weeks through study completion with primary outcome assessment at 8 weeks. The introduction of FYB203 biosimilar to the U.S. market is anticipated in 2023 and in Europe in 2025 [33].

SB-15 (Samsung Bioepis Co. Ltd, South Korea) The initiation of Phase 3 clinical trial for SB15 was announced in June 2020. It is a randomized, double-masked, multicenter study to compare the efficacy, pharmacokinetics, safety, and Immunogenicity between SB15 and original biologic in 446 patients with nAMD. Participants will be randomized 1:1, with each group receiving either SB-15 or Aflibercept every 4 weeks for the first three months, followed by every 8 weeks till Week 48. After week 32, participants in the aflibercept group will be rerandomized into two groups: switch to SB-15 or stay on Aflibercept. The subjects will receive the same dose once every 8 weeks until week 48, followed by a final assessment at week 56. Primary outcome assessment, i.e., change in BCVA, will be done at 8 weeks. The study is scheduled for completion in February 2022 [34].

SOK583A19 (Sandoz, Switzerland) is currently in Phase 3 clinical trial, which started in May 2021. MYLIGHT is a randomized, double-blind, parallel 2-arm study to compare the efficacy, safety, and pharmacokinetics of SOK583A19 to Eylea in patients with nAMD. 460 participants across 20 countries will be randomized to receive either biosimilar or reference product for 48 weeks. The study is scheduled to be completed by May 2023 [35] (Table 2).

CT-P42 (Celltrion, South Korea) has entered phase 3 clinical trial, which started in Feb 2021 and is expected to be completed by November 2022. It is a randomized, active-controlled, double-masked trial to compare the efficacy and safety of CT-P42 with Eylea in patients with Diabetic Macular Edema. A total of 300 participants will be randomized 1:1 to receive either CT-P42 or reference product, and the primary endpoint is the clinical response in BCVA using the ETDRS (Early Treatment Diabetic Retinopathy Study) chart [36].

ALT-L9 (Alteogen, South Korea) In August 2019, Alteogen began Phase 1 clinical trial to compare the efficacy, safety, and pharmacokinetics of ALT-L9 to Eylea in patients with nAMD. However, this trial has not begun recruiting [37]. The company claims better temperature resistance and longer shelf life of ALT-L9 than Aflibercept [3]. OT-702 (Ocumension Therapeutics/Shandong Boan Biological Technology, China) is in phase 3 clinical trial in China. Phase 1 clinical trial showed that it has a good safety profile with no signs of severe adverse reactions [38].

### Biosimilar to Avastin® (Bevacizumab)

Bevacizumab has many biosimilars that have already been approved. The primary use of this drug is in oncology rather than ophthalmology. However, due to the better cost-effectiveness of Bevacizumab than Ranibizumab, its off-label use in Ophthalmology is increasing [39]. Table 3 lists the biosimilars of Bevacizumab, which are approved for clinical use in Oncology.

**Table 3:** Biosimilars of bevacizumab

*Name of Biosimilar*	*Manufacturer*	*Approval Stage*
Zirabev	Pfizer (USA)	US FDA (2019), EMA (2019)
ABP215 (Mvasi)	Amgen (Thousand Oaks, CA, USA) and Allergan (Dublin, Ireland)	US FDA (2017), EMA (2017)
Cizumab	Hetero (Hyderabad, India)	DGCI (2016)
Bevacirel	Reliance Life Sciences (Mumbai, India)	DGCI (2016)
BCD-021	Biocad (Saint Petersburg, Russia)	Russian Regulatory Body (2015)
mAbxience	mAbxience (Madrid, Spain)	Argentina Regulatory Body (2016)
Krabeva	Biocon (Bangalore, India)	DGCI (2017)
Zybev	Zydus Cadila (Ahmedabad, India)	DGCI (2017)
Abevmy	Mylan Pharmaceuticals (South Africa)	DGC! (2017)
Bevatas	Intas (Ahmedabad, India)	DGCI (2017)

Outlook Therapeutics is developing a new Bevacizumab biosimilar, ONS-5010, accepted by the FDA for an Investigational New Drug application. nAMD patients are also enrolled in phase 3 clinical trial to compare the efficacy of ONS-5010 with Bevacizumab using the PIER dosing regimen [39]. If the intravitreal formulation is approved, the compounding bevacizumab could no longer be used as the justification for the use of compounded version may no longer exist [40]. However, it may lead to a paradoxical rise in the cost of Bevacizumab in Ophthalmology.

### Biosimilar to Humira® (Adalimumab)

The three drugs mentioned above are used very often in ophthalmology. However, in certain patients with noninfectious uveitis, a cost-effective biosimilar of adalimumab can be used. The biosimilars of Adalimubab approved for clinical use are briefly mentioned in Table 4.

**Table 4:** Biosimilars of adalimumab

Name of biosimilar	Manufacturer	Approval stage
Cyltezo	Boehringer Ingelheim (Ingelheim am Rhein, Germany)	US FDA (2017) EMA (2017)
Imraldi(EU)/Hadlima (Korea) (SB5)	Samsung Bioepis (Biogen/Samsung)/ Merck South Korea/USA	EMA (2016) Korea (2017)
Amjevita(US)/Amgevita (EU)/Solymbic(EU)(ABP 501)	Amgen (Thousand Oaks, CA, USA)	US FDA (2016), EMA (2017)
GP2017	Sandoz (Basel, Switzerland)	EMA (2018)
Exemptia (ZRC3197)	Zydus Cadila (Ahmedabad, India)	DGCI (2014)
Adfrar	Torrent Pharmaceuticals (Ahmedabad, India)	DGCI (2016)
Mabura	Hetero Drugs (Ahmedabad, India)	DGCI (2018)

## Biosimilars and Immunogenicity

Even though biotherapeutic drugs have revolutionized the treatment of numerous retinal diseases, there are various reports of these drugs causing immunogenic reactions post-intravitreal injections [41–43]. Most of these reactions were associated with the variability in batches during the production and storage of these drugs [44]. Usually, in the eye, adaptive immune reactions do not occur. Therefore, the reports of immunogenic reactions raise queries about the pathophysiology involved in this immune reaction. Initially, these reactions were thought to be a result of the adjuvants present in the injections [45]. However, as the immune reactions were demonstrated after administration of pure proteins, this theory was rejected, and reactions were thought to be associated with the chimeric non-self-nature of the molecules. This theory was rejected as well when fully-humanized therapeutic proteins also resulted in immunogenic reactions [46]. This entire situation resulted in the discovery of anti-drug antibodies (ADA). It has been hypothesized that they develop against the monoclonal antibodies due to their inherent protein-based structure. The adverse effects of these antibodies are thought to occur because of an anaphylactic reaction to the biotherapeutic drugs resulting in sterile endophthalmitis after intravitreal injections. Ophthalmologists also need to understand that these anti-drug antibodies are more likely to develop after injections of overly complex molecules, thus increasing the chances of adverse drug reactions. The incidence of endophthalmitis in patients has demonstrated this after receiving Aflibercept compared to Ranibizumab or Bevacizumab [41].

## Lessons learnt

It is important to note that there will be a lot of potential biosimilars that will soon enter the market over the next decade. Some lessons that can be learned from the experience of launching Razumab in India are as follows:

Developing countries need biosimilar drugs because compounding pharmacies are extremely rare in these countries and hence provide a massive market for ophthalmic biosimilar producers from all over the world.

Pharmacovigilance, quality control, monitoring are particularly important, along with immunogenicity testing assay before market approval to prevent any batch-specific clusters of adverse drug reactions [47].

## Biobetters

The term biobetters was introduced by Mr. GV Prasad, CEO of Dr. Reddy laboratories. It means a biologic that is considered better than the original molecule in one or more aspects with the same target [48]. Biobetters may be modified chemically and have a different amino acid sequence or purification process, resulting in a better drug with better shelf life or pharmacological effects.

The "biobetter" market has a vast potential to grow exponentially. It has slowly gained popularity, with popular drug manufacturers showing keen interest in developing biobetters. However, the issue is that the cost of research and development required to produce a biobetter is ten times the cost required for manufacturing a biosimilar [3]. Even though biobetters can prove to be expensive in terms of research and development, with a proper target and a highly efficacious original biologic, the chances of a biobetter reaching the production stage are incredibly high. Another advantage is that the duration of research and development of a biobetter is comparatively shorter than an innovator molecule [49]. Biobetters are considered investigational new drugs (IND), i.e., a pharmaceutical company can obtain permission from the FDA to initiate human clinical trials and allow shipment of the experimental drug across state lines before a marketing application of the particular drug has been approved. Therefore, they do not have to wait for patents and market exclusivity to elapse, giving better and quicker financial returns to companies than biosimilars.

### Recent developments in biobetter market

1. Genentech is currently developing a port-based delivery system on Ranibizumab. The goal is improved drug delivery with increased efficacy and a reduction in overall treatment cost [50].
2. Ildong pharmaceuticals are also working on a biobetter based on Ranibizumab to improve efficacy and reduce drug resistance in patients receiving Ranibizumab for age-related macular degeneration [51].

3. A Korean-based drug manufacturer, Alteogen, has managed to patent a biosimilar to aflibercept formulation Eylea (produced by Regeneron). Even though this drug has been marketed as a biosimilar, it has an improved shelf life and heat resistance owing to a better manufacturing process [52, 53].

## Conclusion

The world of Ophthalmology, especially Retinal disease management, will witness a massive change in the years to come, as more and more biosimilars are being approved for clinical use from various parts of the globe. These biosimilars are financially rewarding and have tremendous potential due to the multiple available options, economic rates, and prospective coverage of a wider population. While the Biobetter market is still in the developing phase, it has enormous potential to grow. As these are expected to be better than the available biosimilars and the reference molecule by their better formulation and dosing schedule, Biobetters have the potential to revolutionize the posterior segment pharmacotherapy worldwide.

## Data Availability

All data generated or analyzed during this study are included in this published article [and its supplementary information files.
